# A Fluorescent Cell-Based System for Imaging Zika Virus Infection in Real-Time

**DOI:** 10.3390/v10020095

**Published:** 2018-02-24

**Authors:** Michael J. McFadden, Aaron Mitchell-Dick, Christine Vazquez, Allison E. Roder, Kevin F. Labagnara, John J. McMahon, Debra L. Silver, Stacy M. Horner

**Affiliations:** 1 Department of Molecular Genetics & Microbiology, Duke University Medical Center, Durham, NC 27710, USA; michael.mcfadden@duke.edu (M.J.M.); aaron.mitchell.dick@duke.edu (A.M.-D.); christine.vazquez@duke.edu (C.V.); allison.roder@duke.edu (A.E.R.); kevin.labagnara@duke.edu (K.F.L.); mcmahonjohnj@gmail.com (J.J.M.); debra.silver@duke.edu (D.L.S.); 2 Departments of Cell Biology and Neurobiology, Duke University Medical Center, Durham, NC 27710, USA; 3 Department of Medicine, Duke University Medical Center, Durham, NC 27710, USA

**Keywords:** Zika virus (ZIKV), NS2B-NS3, NS4B-NS5, reporter, fluorescence, live cell imaging, apoptosis

## Abstract

Zika virus (ZIKV) is a re-emerging flavivirus that is transmitted to humans through the bite of an infected mosquito or through sexual contact with an infected partner. ZIKV infection during pregnancy has been associated with numerous fetal abnormalities, including prenatal lethality and microcephaly. However, until recent outbreaks in the Americas, ZIKV has been relatively understudied, and therefore the biology and pathogenesis of ZIKV infection remain incompletely understood. Better methods to study ZIKV infection in live cells could enhance our understanding of the biology of ZIKV and the mechanisms by which ZIKV contributes to fetal abnormalities. To this end, we developed a fluorescent cell-based reporter system allowing for live imaging of ZIKV-infected cells. This system utilizes the protease activity of the ZIKV non-structural proteins 2B and 3 (NS2B-NS3) to specifically mark virus-infected cells. Here, we demonstrate the utility of this fluorescent reporter for identifying cells infected by ZIKV strains of two lineages. Further, we use this system to determine that apoptosis is induced in cells directly infected with ZIKV in a cell-autonomous manner. Ultimately, approaches that can directly track ZIKV-infected cells at the single cell-level have the potential to yield new insights into the host-pathogen interactions that regulate ZIKV infection and pathogenesis.

## 1. Introduction

The recent spread of Zika virus (ZIKV) through the Americas has brought renewed attention to the biology and pathogenesis of ZIKV infection [1]. ZIKV clinical manifestations are typically similar to those of other mosquito-borne flaviviruses. These include fever, arthralgia, conjunctivitis, rash, and headache [2]. However, ZIKV infection can lead to more severe pathological outcomes, such as Guillain-Barre syndrome in adults [3,4] and a number of malformations in newborns, collectively termed congenital Zika syndrome [5]. Indeed, the emergence of ZIKV in the Americas coincided with a sharp increase in the number of reported microcephaly cases in Brazil in 2015 [6]. Additionally, numerous biological studies have now associated ZIKV infection with microcephaly and prenatal lethality in animal models [7,8,9,10,11,12]. These and other studies of ZIKV infection in the developing brain of murine models demonstrate the deleterious effects of infection upon neuronal and glia populations [7,10,13,14,15,16,17,18]. In particular, it is now clear that ZIKV infects embryonic neural progenitor cells, resulting in apoptosis and microcephaly [10,15,16,17,18,19]. While the molecular mechanisms underlying the development of microcephaly following ZIKV infection remain incompletely understood, ZIKV-mediated induction of apoptosis is likely an important contributor. The interplay of viral and cellular processes leading to ZIKV-induced apoptosis are also not well understood. Therefore, better tools to study these processes will aid in our understanding of ZIKV pathogenesis and could help with the development of therapeutics.

Current methods to identify ZIKV-infected cells rely on either antibody staining techniques or single-molecule fluorescence, both requiring fixation and permeabilization; or recombinant reporter infectious clones of ZIKV. Fixation precludes the study of ZIKV-infected cells in real-time, and fluorescent reporter flaviviruses, including ZIKV, are generally unstable and attenuated [20,21,22,23]. Experimental tools to identify individual live cells infected with ZIKV could enable new understanding into the cellular processes that underlie ZIKV infection and pathogenesis. Here, we have developed a plasmid-based reporter to specifically identify and track the fate of live ZIKV-infected cells using fluorescence microscopy. This reporter, called ZIKV-nuclear localization signal (NLS)-green fluorescent protein (GFP) (ZIKV-NLS-GFP), encodes the viral non-structural protein 4B and 5 (NS4B-NS5) polyprotein junction upstream of GFP containing a nuclear localization signal (NLS). We show that in ZIKV-infected cells the viral protease, composed of nonstructural proteins NS2B and NS3 (NS2B-NS3), cleaves the reporter NS4B-NS5 polyprotein junction, resulting in nuclear translocation of GFP, thereby indicating infection in real-time. We further demonstrate the utility of this ZIKV-NLS-GFP reporter to identify and monitor ZIKV-infected cells. We show that two strains of ZIKV, representative of both the Asian and African lineages, can cleave the ZIKV-NLS-GFP reporter, and that this system is insensitive to the closely related Dengue virus protease. Finally, we distinguish cell autonomous effects of intracellular ZIKV infection from the effects of paracrine cytokine signaling, finding that ZIKV-infected cells in cell culture more frequently undergo apoptosis than uninfected neighboring cells. Ultimately, this reporter could be effective to use in neural systems and animal models to reveal new insights into cell biological effects of ZIKV infection during pathogenesis.

## 2. Materials and Methods

### 2.1. Cell Lines

Human hepatoma Huh7 cells [24], lung carcinoma A549 cells [25], Vero, and embryonic kidney 293T cells were grown in Dulbecco’s modification of Eagle’s medium (DMEM; Mediatech, Manassas, VA, USA) supplemented with 10% fetal bovine serum (Thermo Scientific, Waltham, MA, USA), 1× minimum essential medium (MEM) non-essential amino acids (Thermo Scientific), and 25 mM HEPES (4-(2-hydroxyethyl)-1-piperazineethanesulfonic acid) (Thermo Scientific) (cDMEM). The identity of the Huh7 cells used in this study was verified by using the GenePrint STR kit (Promega, Madison, WI, USA) (DNA Analysis Facility, Duke University, Durham, NC, USA). A549, 293T, and Vero cells (CCL-185, CRL-3216, and CCL-81) were obtained from American Type Culture Collection (ATCC, Manassas, VA, USA). All cell lines were verified as mycoplasma free by the LookOut Mycoplasma PCR detection kit (Sigma, St. Louis, MO, USA).

### 2.2. Plasmids and Transfections

After reverse transcription (iScript Reverse Transcription Supermix; BioRad, Hercules, CA, USA) to generate complementary DNA from cell lysates infected with ZIKV-Puerto Rico (PR) (PRVABC59; GenBank accession # KU501215), the ZIKV-NLS-GFP reporter plasmid was constructed by first amplifying the nucleotide sequence of ZIKV NS4B and the first ten amino acids of NS5 using Primer 1 and Primer 2 (Table 1). Then, this product was inserted into the p4B5-enhanced green fluorescent protein-dengue virus (p4B5-EGFP DENV) reporter [26], generously provided by Dr. Carey Medin, at the HindIII and EcoRI restriction sites, to generate a p4B5-EGFP ZIKV reporter plasmid, containing a NLS between NS4B-NS5 and EGFP. This construct was subsequently used to amplify the ZIKV NS4B-NS5-NLS-EGFP sequence using Primer 3 and Primer 4 (Table 1). The PCR product was then inserted into the pTRIP vector [27] between the BamHI and XhoI restriction sites, to obtain pZIKV-NLS-GFP. The non-cleavable pZIKV-NLS-GFP RR-AA plasmid was generated by performing site-directed mutagenesis (QuikChange Lightning kit; Stratagene, San Diego, CA, USA) to substitute arginine residues at positions 2519 and 2520 of ZIKV-PR with alanine using Primer 5 and Primer 6 (Table 1). FLAG-tagged ZIKV NS2B-NS3 protease was constructed using the pEF-Tak expression vector [28]. ZIKV NS2B-NS3 sequence was PCR amplified from ZIKV-PR-infected cell lysates using Primer 7 and Primer 8 (Table 1), and ligated into pEF-Tak using the NotI and PmeI restriction sites. The S135A protease dead mutant was generated by performing site-directed mutagenesis on this plasmid using Primer 9 and Primer 10 (Table 1). All DNA sequences were verified by DNA sequencing. DNA transfections were done using FuGENE 6 (Promega).

### 2.3. Generation of Stable ZIKV-NLS-GFP Reporter Cell Lines

Lentiviral particles expressing the ZIKV-NLS-GFP reporter were generated by harvesting supernatant 72 h post transfection of 293T cells with pZIKV-NLS-GFP and the packaging plasmids psPAX2 and pMD2.G (provided by Duke Functional Genomics Facility). This supernatant was then used to transduce A549 cells for 48 h. Following transduction, cells were selected in 2 μg/mL puromycin for 48 h and then serially diluted to obtain single cell GFP-positive colonies. These clones were maintained in cDMEM containing 1 μg/mL puromycin.

### 2.4. ZIKV and DENV-2 Infection

ZIKV-PR (Zika virus/Homo sapiens/PRI/PRVABC59/2015) (GenBank accession #KX087101.3) and ZIKV-Dakar (DAK; Zika virus/A.africanus-tc/SEN/1984/41525-DAK) (GenBank accession #KU955591) were provided by Dr. Mehul Suthar at Emory University and Dr. Scott Weaver at University of Texas Medical Branch, respectively, and stocks were prepared as described [29]. Dengue virus 2 strain 16681 (DENV-2; GenBank accession #KU725663.1) was provided by Dr. Mariano Garcia-Blanco at the University of Texas Medical Branch, and stocks were prepared as described [30]. All viruses were titered by focus forming assay in Vero cells [30]. ZIKV infections were performed in serum-free media conditions for 2 h, after which cDMEM was replenished.

### 2.5. Microscopy

Cells were fixed in 4% paraformaldehyde in phosphate buffered saline (PBS), permeabilized with 0.2% Triton X-100 in PBS, and blocked with 3% bovine serum albumin (BSA) in PBS. Slides were stained with antibodies including rabbit anti-TRAP-α (rabbit antiserum was generated by immunization with keyhole limpet hemocyanin (KLH)-synthetic peptide conjugates and was characterized in prior reports from the laboratory of Dr. Chris Nicchitta at Duke University [31,32], 1:100), rabbit anti-FLAG (Sigma, 1:500), rabbit anti-cleaved Caspase-3 (Cell Signaling Technology, Danvers, MA, USA, 1:1000) or the mouse anti-4G2 antibody (1:500), generated from the D1-4G2-4-15 hybridoma cell line against flavivirus Envelope protein (ATCC). Following primary antibody incubation for 2 h, slides were washed 3× with PBS, and immunostained with conjugated AlexaFluor secondary antibodies (Life Technologies, Carlsbad, CA, USA, 1:500), along with DAPI (4′,6-diamidino-2-phenylindole) (Life Technologies, 1:500), and mounted with ProLong Gold (Invitrogen, Carlsbad, CA, USA).

Confocal microscopy was performed on a Leica SP5 (Wetzlar, Germany) inverted confocal microscope using a 40×/1.25 oil objective using 405, 488, 561 and 633 laser lines at a 4× optical zoom with pinholes set to 1 Airy unit for each channel (Light Microscopy Core Facility, Duke University). Gain and offset settings were optimized and final images were taken with line averaging of 4. Fluorescence microscopy was performed on a Leica DM4B microscope equipped with a Leica DFC 3000 G camera using a 63×/1.40 oil objective using 400, 495, and 585 fluorescence channels. All images were processed with NIH Fiji/ImageJ [33].

### 2.6. Live Cell Imaging and Analysis

A549 cells stably expressing ZIKV-NLS-GFP were plated on glass bottom 12-well plates (MatTek, Ashland, MA, USA; P12G-1.5-14-F). Cells were infected with ZIKV-PR or ZIKV-DAK at multiplicity of infection (MOI) 10 or mock infection at time point 0 h. The culture plate was placed onto the stage of a Zeiss Axio Observer Z.1 equipped with a Pecon incubation chamber (XL multi S1), CO_2_ module S, temperature module S, and humidity control. Cells were incubated at a stable 37 °C with 5% CO_2_. Using Zen software (Zen Blue 2012 1.1.2.0, Carl Zeiss Microscopy GmbH, Oberkochen, Germany), differential interference contrast (DIC) and green fluorescent protein (GFP) immunofluorescence images were acquired at 20× magnification every 10 minutes. Multiple fields were imaged over the time course. Data analysis was performed in Zeiss ZEN and NIH Fiji/ImageJ software.

To calculate the fraction of nuclear GFP cells in a field, sequential images of ZIKV-infected cell cultures were monitored over time to observe translocation of GFP from the cytoplasm to the nucleus of individual cells. The mean fraction of GFP cells was calculated as the number of cells with nuclear GFP over the total number of fluorescent cells per field. Analysis was performed using Zeiss ZEN and NIH Fiji/ImageJ software.

To calculate the time from nuclear GFP translocation to apoptosis, sequential images of ZIKV-infected cell cultures were monitored over time. The time point at which translocation of GFP from the cytoplasm to the nucleus in individual cells was noted, after which the time point of cell death was determined by assessing cellular morphology changes seen in the DIC channel. The time between nuclear GFP translocation and apoptosis was then calculated. In the rare cases that apoptosis was observed without preceding nuclear GFP translocation, the time between the first nuclear translocation event of any cell in the field and the time of apoptosis of the uninfected cell was calculated.

### 2.7. Immunoblotting

Cells were lysed in a modified radioimmunoprecipitation assay (RIPA) buffer (10 mM Tris (pH 7.5), 150 mM NaCl, 0.5% sodium deoxycholate, and 1% Triton X-100) supplemented with protease inhibitor cocktail (Sigma) and phosphatase inhibitor cocktail II (Millipore, Burlington, MA, USA), and post-nuclear lysates were harvested by centrifugation. Quantified protein (between 5 and 15 µg) was added to a 4× SDS protein sample buffer (40% glycerol, 240 mM Tris-HCl (pH 6.8), 8% SDS, 0.04% bromophenol blue, 5% beta-mercaptoethanol), resolved by SDS/PAGE, transferred to nitrocellulose membranes in a 25 mM Tris-192 mM glycine-0.01% SDS buffer, and blocked in StartingBlock buffer (Thermo-Fisher). Membranes were incubated with primary antibodies for 2 h at room temperature. After washing with PBS-T buffer (1× PBS, 0.05% Tween 20), membranes were incubated with species-specific horseradish peroxidase-conjugated antibodies (Jackson ImmunoResearch, West Grove, PA, USA, 1:5000) followed by treatment of the membrane with Clarity enhanced chemiluminescence (ECL) (BioRad) and imaging on X-ray film. The following antibodies were used for immunoblot: rabbit anti-GFP (Thermo-Fisher, 1:5000), rabbit anti-ZIKV NS3 (GeneTex, Irvine, CA, USA 1:5000), rabbit anti-DENV NS3 (GeneTex, 1:5000), anti-FLAG M2 (Sigma, 1:5000), and mouse anti-Tubulin (Sigma, 1:5000).

### 2.8. Nuclear:Total GFP Quantification

Nuclear to total GFP was calculated using a ratio of the mean brightness of GFP in the nuclear area as compared to mean brightness of GFP in the entire cell, as determined using NIH Fiji/ImageJ [33]. For each cell, the area of the nucleus was selected using the DAPI channel and this area was superimposed on the GFP channel. Mean brightness of GFP was measured in the nuclear area alone, and separately, in the total cell (nucleus and cytoplasm). The mean brightness of GFP in the nucleus, divided by the mean brightness of GFP in the total cell is represented as Nuc:Total GFP. Each dot represents an individual cell, where *n* = 18.

### 2.9. Percent of ZIKV+ Cells Quantification

Cells were immunostained for ZIKV Envelope (Env, mouse anti-4G2) and nuclei (DAPI), and cells were identified as ZIKV+ or uninfected by counting 4G2 positive cells using a Cellomics ArrayScan VTI High Content Screening Reader (Duke Functional Genomics Facility, Durham, NC, USA). Percent of ZIKV+ cells was calculated as the number of ZIKV+ cells/the number of total cells (4G2/DAPI) per field. Values represent the mean ± standard error of the mean (SEM) (*n* = 3 fields) from three independent experiments, with >3000 cells counted per field.

## 3. Results

### 3.1. A Cleavable GFP Reporter to Identify ZIKV-Infected Cells

**Figure 1:**
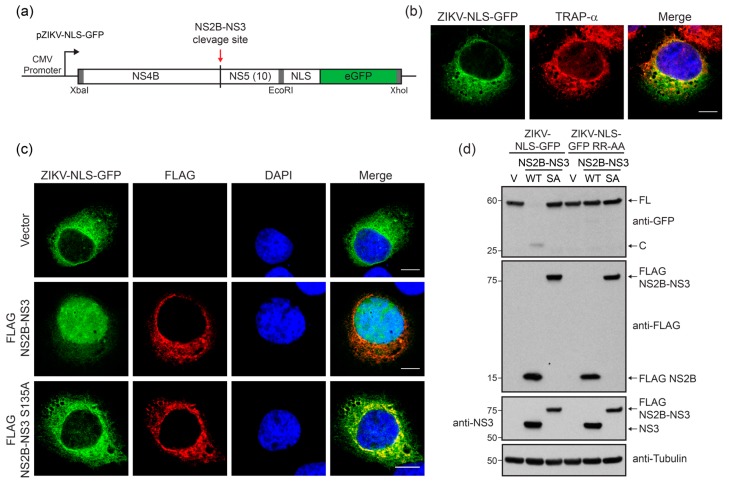
A cleavable reporter to measure Zika virus (ZIKV) non-structural proteins 2B and 3 (NS2B-NS3) protease cleavage. (**a**) Schematic of the fluorescent ZIKV-nuclear localization signal (NLS)-GFP reporter plasmid (pZIKV-NLS-GFP) construct encoding ZIKV non-structural protein 4B (NS4B) (aa2270–2520) and the first 10 amino acids of non-structural protein 5 (NS5) (aa2521–2530), fused in frame to a nuclear localization signal (NLS) and enhanced green fluorescent protein (eGFP). The red arrow indicates the NS2B-NS3 protease cleavage site. Restriction sites used for cloning are indicated by gray boxes. (**b**) Confocal micrographs of Huh7 cells expressing ZIKV-NLS-GFP (green) and immunostained with the endoplasmic reticulum (ER) marker translocon-associated protein, alpha subunit (TRAP-α) (red). Nuclei were stained with DAPI (4′,6-diamidino-2-phenylindole) (blue). Scale bar, 10 µm. (**c**) Confocal micrographs of Huh7 cells expressing ZIKV-NLS-GFP (green) and either FLAG-tagged-NS2B-NS3, WT or S135A, or vector, that were immunostained with anti-FLAG (red). Nuclei were stained with DAPI (blue). Scale bar, 10 µm. (**d**) Immunoblot analysis of extracts from Huh7 cells expressing either WT ZIKV-NLS-GFP or a non-cleavable ZIKV-NLS-GFP RR-AA reporter, and also either wild-type (WT) or S135A (SA) FLAG-tagged ZIKV NS2B-NS3, or vector (V). Arrows mark full-length (FL) or cleaved (C) ZIKV-NLS-GFP.

To monitor cells infected by ZIKV in real-time, we constructed a reporter plasmid (ZIKV-NLS-GFP) that encodes the ZIKV NS4B protein and the first ten amino acids of NS5, as well as a NLS upstream of GFP, in a similar strategy to those previously employed for hepatitis C virus and dengue virus [26,34] (Figure 1a). Like all flaviviruses, ZIKV encodes a polyprotein that is processed by both host and viral proteases, including NS2B-NS3, into the individual proteins of the virus [35,36]. Therefore, upon ZIKV infection, we would expect that cleavage of the junction between NS4B and NS5 by the viral NS2B-NS3 protease would release NLS-GFP from the endoplasmic reticulum (ER) tether for trafficking to the nucleus. Because ZIKV NS4B localizes to the ER membrane, we first determined the localization of the transfected reporter in uninfected human hepatoma Huh7 cells by using immunostaining and confocal microscopy. We found that the GFP fusion protein colocalized with the ER membrane protein translocon-associated protein, alpha subunit (TRAP-α) [37] in Huh7 cells expressing the reporter (Figure 1b). Expression of a wild-type (WT) FLAG-tagged ZIKV NS2B-NS3 protease resulted in nuclear translocation of GFP, while expression of the protease inactive (SA) NS2B-NS3 S135A mutant did not (Figure 1c). Immunoblot analysis of lysates from transfected cells confirms that while expression of inactive NS2B-NS3 SA protease did not cleave the ZIKV-NLS-GFP reporter, expression of NS2B-NS3 WT protease resulted in cleavage of the ZIKV-NLS-GFP reporter into the expected products of 56 kD and 29 kD (Figure 1d). Importantly, inactivation of the protease cleavage site in the reporter by alanine substitution of the dibasic arginine residues prevented cleavage by the expressed NS2B-NS3 protein (Figure 1d). Together, these data indicate that the protease activity of ZIKV NS2B-NS3 is necessary for site-specific cleavage of the GFP reporter and its translocation to the nucleus.

### 3.2. The Cleavable ZIKV-NLS-GFP Reporter Can Detect ZIKV Infection

**Figure 2:**
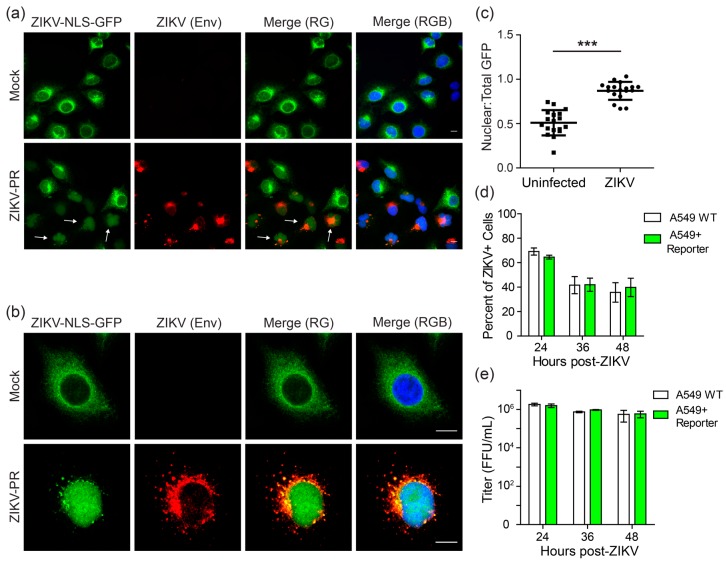
The cleavable ZIKV-NLS-GFP reporter can detect ZIKV infection. (**a**,**b**) Fluorescence (**a**), or confocal micrographs (**b**) of A549 cells stably expressing the ZIKV-NLS-GFP reporter (green) that were infected with ZIKV-PR (multiplicity of infection (MOI) 1) or mock for 48 h and then immunostained for ZIKV (anti-Envelope (Env); red). Nuclei were stained with DAPI (blue). Scale bar, 10 µm. Arrowheads in the bottom panel of (**a**) indicate some representative cells staining positive for ZIKV with nuclear GFP localization. (**c**) The ratio of nuclear to total fluorescence intensity, as determined using Image J, in uninfected and ZIKV-infected cells from confocal micrographs of cells immunostained for ZIKV. Each dot represents an individual cell (*n* = 18). Dot plots represent the mean ± SD. Asterisks denote significance; *** *p* < 0.0001, as determined by an unpaired *t*-test. (**d**) Fields were captured at various time points post-infection with ZIKV-PR (MOI 10) of A549 cells (WT or stably expressed ZIKV-NLS-GFP) immunostained for ZIKV Env and nuclei (DAPI), and the percentage of ZIKV+ cells was calculated. Values represent the mean ± standard error of the mean (SEM) (*n* = 3 fields) from three independent experiments, with >3000 cells counted per field. (**e**) Supernatants from A549 cells (WT or stably expressed ZIKV-NLS-GFP) were harvested at 24, 36, and 48 h post-infection with ZIKV-PR (MOI 10) and viral titer was measured by focus forming assay. Values represent the mean ± standard deviation (SD) (*n* = 3) from one experiment, representative of two independent experiments.

We next determined the functionality of the reporter during ZIKV infection. For these assays, we used A549 cell clones that had been transduced with a lentivirus encoding the ZIKV-NLS-GFP reporter. Importantly, A549 cells have been shown previously to support ZIKV replication [38]. In ZIKV-NLS-GFP-expressing A549 clonal cell lines, uninfected cells exhibit cytoplasmic GFP staining, consistent with that seen in Figure 1 (Figure 2a,b, top panels). However, following infection with the Puerto Rican strain of ZIKV (ZIKV-PR), microscopy revealed nuclear translocation of GFP in infected cells (Figure 2a,b, bottom panels). Further, unbiased quantification of individual cells across multiple microscopy fields showed a significant increase in the overall ratio of nuclear GFP to total GFP fluorescence during ZIKV infection (Figure 2c). Importantly, we also found that these A549 cells stably expressing the ZIKV-NLS-GFP reporter supported ZIKV replication to a similar level as the parental A549 cells, as determined by the number of cells that stained positive for ZIKV Envelope protein (Env) at 24, 36, and 48 h post-infection with ZIKV-PR (Figure 2d). Further, these cells also produced similar levels of infectious virus as parental cells, as measured by viral titer in the supernatant at 24, 36, and 48 h post-infection with ZIKV-PR (Figure 2e). These data indicate that cell lines expressing the ZIKV-NLS-GFP reporter can be used to identify ZIKV-infected cells without affecting viral replication. Additionally, these results suggest that this fluorescent cell-based system could be used to monitor ZIKV-infected cell cultures in real-time.

### 3.3. Two Divergent Strains of ZIKV Can Both Cleave the ZIKV-NLS-GFP Reporter

**Figure 3:**
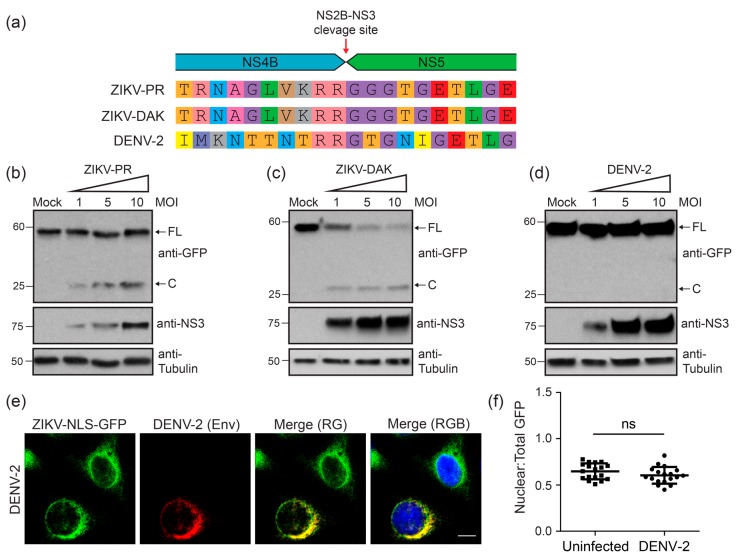
Two divergent strains of ZIKV can both cleave the ZIKV-NLS-GFP reporter. (**a**) Alignment of viral amino acids adjacent to the NS2B-NS3 cleavage site (amino acids 2511–2530 of ZIKV and 2482–2501 of DENV-2), including the Puerto Rico (ZIKV-PR) and Dakar (ZIKV-DAK) ZIKV strains, as well as dengue virus serotype 2 (DENV-2). (**b**–**d**) Immunoblot analysis of extracts from A549 cells stably expressing ZIKV-NLS-GFP that were mock-infected or infected with an increasing MOI (1, 5, or 10) of ZIKV-PR (**b**), ZIKV-DAK (**c**), or DENV-2 (**d**). Arrows mark full-length (FL) or cleaved (C) ZIKV-NLS-GFP reporter. (**e**) Confocal micrographs of A549 cells stably expressing the ZIKV-NLS-GFP reporter (green) that were infected with DENV-2 (MOI 10) for 48 h and then immunostained for DENV-2 (anti-Envelope (Env); red). Nuclei were stained with DAPI (blue). Scale bar, 10 µm. (**f**) The ratio of nuclear to total fluorescence intensity, as determined using Image J, in uninfected and DENV-2-infected cells immunostained for DENV Env. Each dot represents an individual cell (*n* = 18). Dot plots represent the mean ± SD. (ns) denotes *p* > 0.05, as determined by an unpaired *t*-test.

To determine if the ZIKV-NLS-GFP reporter could be used for multiple strains of ZIKV, we tested its utility in monitoring infection by the African lineage Dakar strain of ZIKV (ZIKV-DAK). Notably, the ten amino acids immediately up- or down-stream of the NS4B-NS5 protease cleavage junction are highly conserved between the African and Asian ZIKV lineages (Figure 3a), as well as in more than 50 ZIKV strains examined from the Virus Pathogen Resource database. However, these residues are not fully conserved in the closely related dengue virus serotype 2 (DENV-2) (Figure 3a). To demonstrate that both Asian and African lineage strains of ZIKV result in cleavage of the ZIKV-NLS-GFP reporter, we infected cells with an increasing multiplicity of infection (MOI) of either the Puerto Rican (ZIKV-PR) or Dakar (ZIKV-DAK) strains and measured cleavage by immunoblot analysis of cell lysates at 24 h post-infection. We found that the reporter was cleaved in a viral dose-responsive fashion, as measured by the presence of the 29 kD cleavage product in infected cell lysates (Figure 3b,c). Further, we found that the ZIKV-NLS-GFP reporter was not cleaved in cells infected with DENV-2, even at a high MOI (Figure 3d). The inability of DENV-2 to cleave the ZIKV-NLS-GFP reporter was confirmed by immunofluorescence analysis and quantification (Figure 3e,f). Therefore, the ZIKV-NLS-GFP reporter is sensitive to cleavage by ZIKV strains of both the Asian and African lineages, but not by the more divergent DENV-2.

### 3.4. Live Cell Imaging Determines Kinetics of Nuclear Localization of GFP Following ZIKV Infection

**Figure 4:**
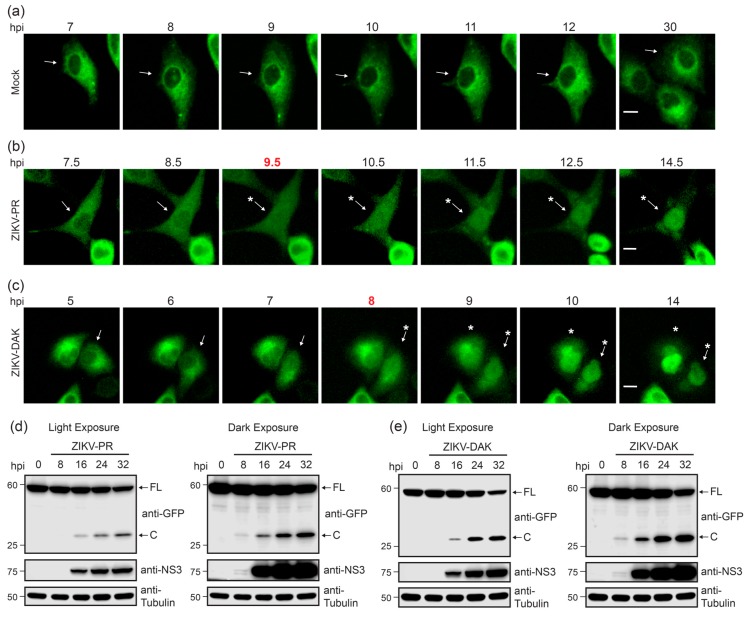

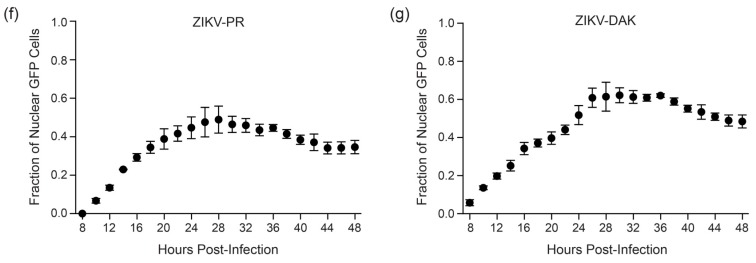
Kinetics of nuclear localization of GFP following ZIKV infection. (**a**–**c**) Fluorescence microscopy shows sequential images of the same field of A549 cells stably expressing ZIKV-NLS-GFP after infection (MOI 10) with mock (**a**), ZIKV-PR (**b**) or ZIKV-DAK (**c**) at the indicated hours post infection (hpi) above each image. Red, bolded time points represent the first time at which nuclear GFP could be detected. Arrows mark the cell of interest and asterisks indicate cells with nuclear GFP. Scale bar, 10 µm. (**d**,**e**) Immunoblot analysis of extracts from A549 cells stably expressing the ZIKV-NLS-GFP reporter that were mock-infected or infected with ZIKV-PR (MOI 10) (**d**), or ZIKV-DAK (MOI 10) (**e**) for the indicated hours (hpi). Arrows mark full-length (FL) or cleaved (C) ZIKV-NLS-GFP, with both light and dark exposures of indicated blots shown. (**f**,**g**) Quantification of the experiments in (**b**,**c**) in which the number of cells positive for ZIKV in a field were identified by determining the incidence of GFP nuclear translocation in each cell at various time points following ZIKV infection. Fractions represent the number of cells with nuclear GFP over the total number of fluorescent cells in a field. Values represent the mean ± SEM (*n* = 5 fields) from three independent experiments, with >20 cells counted per field.

We next sought to determine the kinetics of nuclear translocation of GFP from the ZIKV-NLS-GFP reporter during infection using live cell imaging. As expected, we did not observe nuclear translocation of GFP in mock-infected cultures (Figure 4a and Movie S1). Alternatively, GFP nuclear translocation was first detected in individual cells as early as 9.5 h post-ZIKV-PR infection and nearly complete nuclear translocation of GFP was observed by 12.5 h post-infection (Figure 4b). Similarly, following ZIKV-DAK infection, GFP nuclear translocation in individual cells was visible by 8 h post-infection, and nearly complete GFP nuclear translocation was first observed by 10 h post-infection (Figure 4c). This time frame corresponded to expression of the viral NS3 protein during both ZIKV-PR and ZIKV-DAK infection and cleavage of the reporter, detected as early as 8 h post-infection, as measured by immunoblotting (Figure 4d,e). Additionally, cleavage increased concordantly with viral replication over time (Figure 4d,e). These experiments suggest that ZIKV-DAK replicates slightly more efficiently than ZIKV-PR in our system. On a population level, the fraction of cells with nuclear GFP over the total number of fluorescent cells per field increased within the first 28 h. This increase was then followed by a plateau in the fraction of cells with nuclear GFP (Figure 4f,g) and ultimately their death (Movies S2 and S3). Additionally, live imaging of ZIKV-infected A549 cells stably expressing the ZIKV-NLS-GFP reporter allows tracking of infected cells following initial nuclear translocation of GFP, even if the nuclear signal subsequently weakens because of decreased expression or stability (Movies S2 and S3). Thus, the ZIKV-NLS-GFP reporter can be used to identify and track individual ZIKV-infected cells in real-time.

### 3.5. ZIKV Induces Cell Death Specifically in Infected Cells

**Figure 5:**
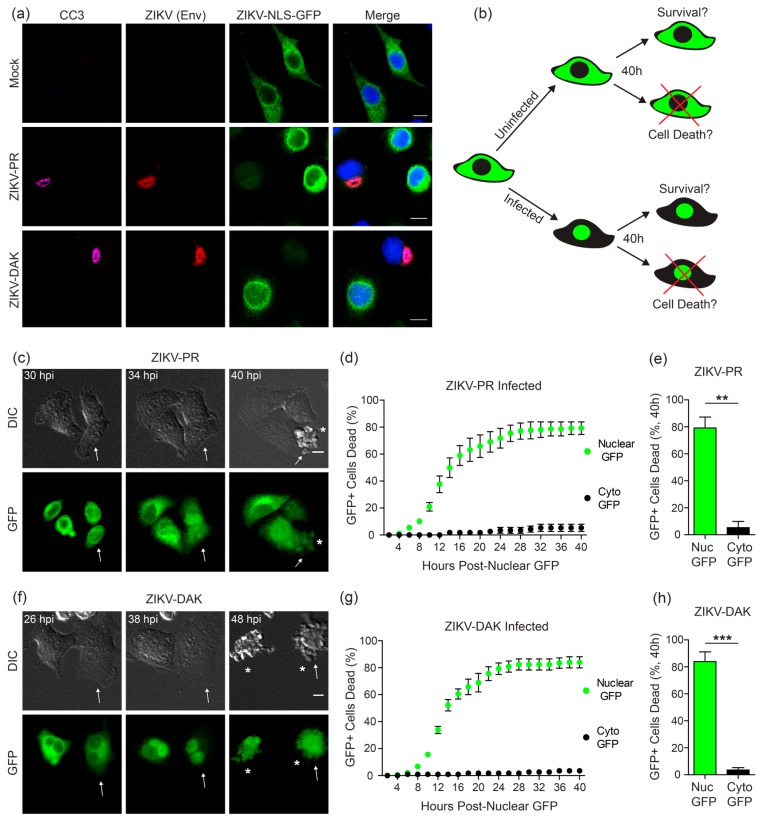
ZIKV induces cell death in virus-positive cells. (**a**) Confocal micrographs of A549 cells stably expressing the ZIKV-NLS-GFP reporter (green) that were infected with ZIKV-PR or ZIKV-DAK (MOI 10), or mock for 48 h and then immunostained for ZIKV (anti-Envelope (Env); red) and cleaved Caspase-3 (CC3; magenta). Nuclei were stained with DAPI (blue). Scale bar, 10 µm. (**b**) Schematic of the live cell imaging analysis performed to quantify cell death in ZIKV-uninfected and infected cells. (**c**,**f**) Fluorescence microscopy shows sequential images of the same field of A549 cells stably expressing ZIKV-NLS-GFP after infection (MOI 10) with ZIKV-PR (**c**) or ZIKV-DAK (**f**) at the indicated hours post infection (hpi) between corresponding DIC (top) and GFP (bottom) images. Arrows mark cells of interest and asterisks denote cells that have died. Scale bar, 10 µm. (**d**,**e**,**g**,**h**) Quantification of the time of death of cells in ZIKV-infected cultures from live cell imaging experiments (represented by Movies S2 and S3). Individual fluorescent cells were tracked and identified as positive for ZIKV by determining the incidence and time point of nuclear GFP translocation. The time point of cell death was determined by assessing cellular morphological changes as seen in the DIC channel, and the time between GFP translocation and cell death was calculated. For uninfected cells that did not undergo nuclear translocation, the time between apoptosis and the first nuclear translocation event in the entire field was calculated and plotted. Values represent the mean ± SEM (*n* = 10 fields) from three independent experiments, with 10 cells counted per field. Cell death in infected (Nuc GFP) and uninfected (Cyto GFP) cells is directly compared at 40 h post-nuclear GFP translocation in (**e**,**h**). Asterisks denote significance; ** *p* = 0.0002 (**e**) and *** *p* < 0.0001 (**h**), as determined by an unpaired *t*-test.

ZIKV infection induces caspase cleavage and apoptotic cell death in A549 cells and in neuronal systems [15,16,17,18,22,39,40]. Indeed, we could detect cleaved Caspase-3 (CC3) in A549 cells stably expressing the ZIKV-NLS-GFP reporter following 36 h of ZIKV infection (Figure 5a). Interestingly, a number of reports have shown that following ZIKV infection, CC3 is detectable in neural cells that are not expressing viral proteins, suggesting that paracrine cytokine signaling is sufficient to induce apoptosis in response to ZIKV infection [16,39,41,42]. However, at least one report showed that CC3 expression was only increased in ZIKV-positive cells in ZIKV-infected human cortical organotypic brain slices [43]. To determine whether apoptosis occurs in uninfected or ZIKV-positive cells in our system, we used fluorescence microscopy to monitor the population of A549 cells stably expressing the ZIKV-NLS-GFP reporter during ZIKV infection to track cell death (Figure 5b). Cells were determined dead by morphological changes in the DIC channel (Figure 5c,f, Movies S1–S3). We observed cell death at a single cell level in cultures that were infected with ZIKV-PR or ZIKV-DAK. For both ZIKV strains, more than 50 percent of cells died within 16 h after cells exhibited nuclear GFP translocation, and only a very small percentage of cells with nuclear GFP survived for the duration of imaging (Figure 5d,g). However, the uninfected cells in the ZIKV-infected cultures, as determined by no nuclear GFP translocation, did not typically undergo cell death (Figure 5d,g). By 40 h post-nuclear translocation of GFP, approximately 80 percent of infected cells had died, whereas less than 10 percent of uninfected cells had died (Figure 5e,h). These data suggest that ZIKV-induced apoptosis in cell culture is dependent upon active intracellular viral replication and is likely not mediated by paracrine cytokine signaling.

## 4. Discussion

Here, we describe a fluorescent cell-based approach for imaging ZIKV infection at a single-cell level in real-time. The ZIKV-NLS-GFP reporter depends on the protease activity of ZIKV NS2B-NS3, which cleaves the reporter polyprotein to trigger nuclear translocation of GFP (Figure 1). This results in specific labeling of infected cells, coinciding with ZIKV protein expression (Figure 4). This reporter is activated by ZIKV strains of both Asian and African lineages, but not by the closely related DENV-2 (Figure 3). Importantly, stable expression of the ZIKV-NLS-GFP reporter in cell cultures is not detrimental to viral infection (Figure 2). By using this reporter, we found that in culture, death occurs in cells with intracellular ZIKV infection and to a much lesser extent in neighboring uninfected cells (Figure 5). Overall, the ZIKV-NLS-GFP reporter can be used to track ZIKV-infected cells in real-time using fluorescence microscopy.

While numerous reports have shown increased apoptosis in neural cell systems following ZIKV infection [10,15,16,17,18,39,40], whether this apoptosis is mediated by paracrine cytokine signaling or direct viral infection is not fully established. Interestingly, in the developing brains of ZIKV-infected mice, some cells were positive for cleaved Caspase-3 in the absence of detectable ZIKV proteins [39,41]. Additionally, in human neural progenitor cell culture, ZIKV infection led to P53 and Caspase-3 activation in some cells without detectable ZIKV proteins [16,42]. However, others have shown that in ZIKV-infected human cortical organotypic brain slices, the ZIKV-positive cells exhibited increased cleaved Caspase-3 expression, while the ZIKV-negative cells in the slices did not [43]. In our system, in which we can sensitively track ZIKV-infected cells without staining for viral proteins, cells with replicating ZIKV undergo higher levels of apoptosis than their neighboring uninfected cells (Figure 5). These findings suggest that ZIKV-mediated apoptosis is not activated by paracrine cytokine signaling in cell culture, but rather occurs in a cell-autonomous manner. Whether uninfected cells undergo apoptosis at a later time point, or how relevant these observations in A549 cells are to neural cells, will require further investigation. Future research utilizing the ZIKV-NLS-GFP reporter system in these additional cell types may reveal the mechanisms by which ZIKV infection induces P53 and caspase activation and apoptosis in diverse cell types or in animal models.

The ability to distinguish infected cells from uninfected cells in real-time by live cell imaging is a particularly useful application of the ZIKV-NLS-GFP reporter. Current strategies for studying how ZIKV infection alters the cellular biology of the developing brain or neural progenitor cells have mostly relied on fixation and antibody staining. While this allows for the identification of individual ZIKV-infected cells, they cannot be observed in real-time. The approach described in this manuscript could be used to study direct effects of intracellular ZIKV infection on cell biological processes, including mitosis, which ZIKV infection can disrupt [17,44,45]. It is possible that the attenuation and instability of fluorescent reporter flaviviruses [20,22] may cause limitations for their use in vivo. Future work to establish animal models expressing the ZIKV-NLS-GFP reporter may help to overcome this limitation. We anticipate that these animal models could aid studies of ZIKV tropism or live cell imaging of ZIKV infection in brain slices from infected animals [45,46]. One major limitation of the ZIKV-NLS-GFP reporter presented here is the inability to use high-throughput cell sorting for downstream analyses as this reporter measures subcellular localization changes that would not be distinguishable by fluorescence-activated cell sorting. Reporters that activate fluorescence specifically in infected cells by taking advantage of the specificity of the viral NS2B-NS3 protease for viral infection will be useful in this regard.

The ZIKV-NLS-GFP reporter provides a unique tool for live cell imaging of ZIKV-infected cell cultures. Future studies utilizing this fluorescent cell-based approach for identification of ZIKV-infected cells in real-time will expand our knowledge of the biology and pathogenesis of ZIKV infection. Potential applications include studying ZIKV tropism, tracking the fate of infected cells in the developing mouse brain and peripheral nervous system, and antiviral drug screening.

## Figures and Tables

**Table 1:** Primers used.

Primer Name	Primer Sequence
Primer 1 (734)	5′-TTAAGCTTGCCACCATGGCGAATGAACTCGGATGGTTGGA-3′
Primer 2 (735)	5′-CATGGTGGCGAATTCCTCTCCCAGGGTCTCTCCTGTTC-3′
Primer 3 (740)	5′-TCTAGAGGATCCGGAAAGCTTGCCACCATGGCGAATGAA-3′
Primer 4 (741)	5′-TTTCTAGGTCTCGAGTTACTTGTACAGCTCGTCCATGCCG-3′
Primer 5 (864)	5′-ACGCTGGCTTGGTCAAGGCAGCTGGGGGTGGAACAGGAG-3′
Primer 6 (865)	5′-CTCCTGTTCCACCCCCAGCTGCCTTGACCAAGCCAGCGT-3′
Primer 7 (880)	5′-ATAAAGCGGCCGCTAGCTGGCCCCCTAGCGAAGT-3′
Primer 8 (881)	5′-AGCGGGTTTAAACTTATCTTTTCCCAGCGGCAAACT-3′
Primer 9 (886)	5′-ATTACCCAGCAGGAACTGCAGGATCTCCAATCCTA-3′
Primer 10 (887)	5′-TAGGATTGGAGATCCTGCAGTTCCTGCTGGGTAAT-3′

## Supplementary Materials

The following are available online at http://www.mdpi.com/1999-4915/10/2/95/s1, Movie S1: Mock, Movie S2: ZIKV-PR, Movie S3: ZIKV-DAK.
